# Duration of Normal Human Pregnancy

**Published:** 1881-04

**Authors:** P. O’Connell

**Affiliations:** Sioux City, Iowa


CHICAGO MEDICAL

JOURNAL & EXAMINER.

Vol. XLII.—APRIL, 1881.—No. 4.

### Original Communications.

Article I.

**Duration of Normal Human Pregnancy.** By P. O'Connell, m.d., Sioux City, Iowa.

From time immemorial, writers on obstetrics and on forensic medicine in particular, have endeavored to determine the duration of utero-gestation in the human female. I should be extremely glad to settle this *questio vexata* in a manner as satisfactory and as convincing to others as I have to myself. But I do not flatter myself with any such notion of success. All I can hope to do is to add a few facts, from personal observation and reflection, towards settling this very important matter. to which my attention was directed, in a special manner, several years ago, when a very intelligent young lady engaged me to attend her in her first labor which she predicted to the very day. This had the effect of inducing me to devote particular care and attention to the investigation of the duration of normal gestation in women coming under my care, with what result I leave others to judge from the cases collected by me, to the number of fourteen, in each of which the fruitful act of intercourse is pretty accurately known. And while endeavoring to show what is the duration of normal human pregnancy, the cases I give will help to settle other questions intimately connected with the matter under discussion.

The following questions are those to which I would draw particular attention, and endeavor to answer; for, it seems to me, that if we could solve them, we could readily determine the duration of utero-gestation:

1. Where does fecundation take place?
2. What time do the spermatozoa take to reach the ovum?
3. What time does the ovum take to descend from the ovary to the uterus?

These three questions are most important, yet exceedingly difficult to answer. The following, while interesting, are of much less consequence:

4. What relation does conception bear to menstruation?
5. Does labor set in exactly at a time to correspond with a tenth recurrence of menstruation if conception had not occurred?
6. Is sex dependent on the number of spermatozoa finding access to the ovum?
7. Is sex dependent on the time of conception relative to menstruation?
8. Is the first child carried to full term?

1. Where does fecundation take place?

A few physiologists have asserted that the ovum loses the power of being fecundated when it reaches the lower end of the fallopian tube; while a few others affirm that it is arrested and developed at the spot where the spermatozoa meet it. Both theories lack proof. Fecundation may take place while the ovum is still in the ovary; or during its transit along the oviduct at any point thereof; or when in the uterus. The two former propositions are proved by abdominal, true ovarian, and by tubal gestation. Fecundation is liable to occur at any spot between the ovary and the *os uteri internum*. But the precise place where it happens, in any given case of uterine gestation, cannot, of course, be determined.

2. What time do the spermatozoa take to reach the ovum?

The answer to this question depends on the place where the sperm cells and germ cell meet; and that, in turn, depends partly on the time of escape of the ovum from the ovary; and partly on the rate of motion upwards of the spermatozoa, and on the rate of motion downwards of the ovum. Bischoff demonstrated the presence of spermatozoa on the ovaries of animals killed a week after the congress of the sexes. Dr. Austin Flint, junior, saw them, *in motion*, on the surface of the ovary eight days after intercourse. Dr. S. R. Percy found them alive in mucus issuing from the os uteri of a woman eight and a half days after coitus. These facts prove that spermatozoids can and do live several days in the female generative tract. Bischoff's and Flint's discoveries demonstrate the distance to which spermatozoa can penetrate, but do not, unfortunately, determine the time they occupied in their upward course to the ovary. And Dr. Percy's discovery shows that although they may enter the uterine canal, they do not necessarily find their way to the ovarium, but are liable to be swept back by excess of secretion (catarrh) from the uterine canal. It is my opinion that spermatozoa may, under favorable conditions, reach the lower end of the uterine cavity; that is, may pass the *os uteri internum* in a few hours. I am also of opinion that they may not reach the ovary for ten or fifteen days after coitus.

3. What time does the ovum occupy in its descent from the ovary to the uterus?

This question is not more definitely settled than either of the two preceding. Physiologists appear to think that eight or ten days are *usually* occupied by the transit of an ovum from the ovary to the uterine cavity. If, as I suppose, the peristaltic motion of the fallopian tube be much more brisk about and during the time of menstruation than during the intermenstrual period, then an ovum may be propelled from the ovary to the uterus in two or three days in the former case; whereas, in the latter, it may require fifteen or sixteen days, as would appear to have happened in the sixth case of my table.

4. What relation does conception bear to menstruation?

They are correlated. I believe menstruation is dependent on ovulation. The maturation and extrusion of an ovum constitute the stimulus to the menstrual flow. Ovulation and menstruation are simply cause and effect. When are ova discharged? They are discharged before and after, but chiefly during, menstruation. Usually the rupture of the Graafian follicle occurs during or about the end of the first day of menstruation. An ovum discharged before the onset of the menses, is generally swept away in the menstrual flood ; when discharged during the flow, it will not reach the uterine cavity until the menses have ceased. This explains the greater frequency of conception following coitus held immediately after menstruation. The ovum fecundated is that which acts as the exciting cause of the menstruation imminent or just ceased. My cases show, also, that conception may take place immediately before as well as immediately after menstruation. When conception occurs just before the outset of the menses, the flow is either entirely arrested or so modified in color and quantity as to attract the woman's attention.

But conception may, and actually does, occur at any time between the menstrual epochs, as is proved by the American women, most of whom practice, I regret to say, the trick of non-submission to coitus for about ten days after menstruation, with the sole and criminal intention of avoiding conception and family, if possible ; yet they are not always successful, as many discover to their surprise and chagrin. It is also proved by the Jewish women who are very prolific, yet are required by the Mosaic law to abstain (being considered unclean) from all intercourse, most especially from coitus, with the entire household for seven to fourteen days after menstruation ; * * * "she shall be unclean two weeks according to the custom of her monthly courses."* "The woman, who, at the return of the month, hath her issue of blood, shall be separated seven days."† Case 6 of my table also illustrates this point, as well as shows how long an ovum may take to descend from the ovary to the uterus. While conception is not confined to the periods of menstruation, it is more likely to occur within a few days after the cessation of the menstrual flow, than at any other time. It is quite certain that there is a greater aptitude for conception, and that females expe-

* Levit. xii, 5.

† Levit. xv, 19.

rience stronger sexual desire, immediately before and after menstruation than at any other time; a fact well known to the ancient physicians, who frequently recommended, and with success, coitus immediately after menstruation as a cure for sterility in women. Raciborski affirms that the exceptions to conception taking place immediately before, during, and immediately after menstruation, are not more than six or seven per cent. "But it is certain that conception is much more likely to take place soon after they" (the menses) "have ceased to flow, or even just before their access, than in the intervening period; so that, in most instances, it would be most correct to expect labor at forty weeks and a few days after the last recurrence of the menses."*

5. Does labor set in exactly at a time to correspond to a tenth recurrence of the menses if conception had not occurred?

"The idea has of late years been put forward and sustained by direct observation, that in women whose menstrual function is regular, gestation will terminate at the tenth menstrual period after that upon which conception has ensued. Then, as the ordinary menstrual interval is about twenty-eight days, the ordinary duration of pregnancy would be a few days less than 280 days, varying according to the time occupied by the monthly flow."† This theory is negatived by cases 6, 7, 9, 11 and 14 of my table.

6. Is sex dependent on the number of spermatozoa finding access to the ovum?

Some writers assert that sex is determined by the number of spermatozoa; that when conception takes place just before menstruation, few spermatozoons enter the ovum, and a female child is born; that after menstruation, many enter and a male child is developed. I fail to see why only a few sperm cells should enter the germ cell before menstruation and many after that act. Where or what is the impediment to prevent as many entering the ovum before menstruation as after it? Cases 3, 4, and 7 of my table do not bear out this theory. Besides, twins at either time, of different sex, flatly contradict it.

The number of spermatozoids finding access to an ovum does

---

* Carpenter's Physiology, New American, from Eighth English Edition, page 910, *Note* 2.

† Wharton and Stillé Medical Jurisprudence, Second Edition, page 312.

not appear to play any part in determining sex. Mr. Newport ascertained that a too copious application of spermatozoa to an ovum is absolutely unfavorable to their action—will not fecundate at all. Others assert, perhaps with truth and reason, that a single sperm cell is sufficient for the purpose of fecundation. I believe very few are necessary. It is well-known that the introduction of a small quantity of seminal fluid just within the *sphincter vaginæ*, or, for the matter of that, on the vulva is all that is necessary, sometimes, for the purpose of impregnation ; as some women have found to their cost. This very point was demonstrated in 1876, when Prof. Braun published three cases of pregnancy with intact hymen. In the first case, the aperture in the hymen barely admitted the uterine sound ; in the third the opening in the hymen was a short and narrow slit. Prof. Braun ascertained that penetration did not take place in the first and third; that the semen was deposited on the hymen. Consequently the number of spermatozoa which could find access to the ovum must have been very limited indeed.

7. Is sex dependent on time of conception relative to menstruation ?

Certainly not, as twins of different sex conceived before or after menstruation prove, and as my cases also show.

8. Is the first child carried to full term ?

Some suppose not ; others think the sex influences the duration of gestation a little ; as for example, a female child is born, or apt to be born, a few days before full term ; and *vice versa* with a male child. This idea does not hold good in either case. No matter what the sex, the first child is carried to full term, as my cases prove.

Finally, I would ask, What is the duration of normal utero-gestation in the human female ? Is it nine calendar months ? Perhaps so ; but nine calendar months may mean 273, 274, 275, or 276 days. Is it ten lunar months, forty weeks, or 280 days ? I do not believe so. Between ten lunar months or forty weeks, and nine calendar months there may be a difference of just one week.

It could serve no purpose beyond occupying valuable space, to heap up tables or collections of cases, as is too often done, giving the minimum, mean and maximum duration of gestation. From all such collections or tables it will simply be seen that the majority of women are delivered between the two hundred and seventy-third and two hundred and eightieth day; that the mean duration of pregnancy is 275 or 276 days. Between the minimum and maximum duration, however, there may be the very great difference of forty days! "In a practical point of view, we may consider that the average duration of pregnancy is * * * about 274 or 275 days from the time of coitus, when this can be ascertained."*

The opinion entertained and long pondered over by me is, that the duration of normal utero-gestation in the human female is 275 days from the time the fecundated ovum takes up its place in the uterus to the outset of labor. Besides the cases coming under my personal observation, here given in the table, and strongly sustaining my opinion, I have been led to this conclusion on what, to many, may, perhaps does, seem inadequate data, but which to me appear absolutely irresistible and convincing; I mean the duration of the intra-uterine life of Christ, which was exactly 275 days or nine calendar months. We have His own words in testimony that He did not come to destroy nor to subvert the law, but to fulfill it to the very letter, which He did in every manner. I have often wondered, but am unable to explain, why it is I had never seen this fact mentioned in the chapters of works on obstetrics and forensic medicine where the attempt is made to solve the duration of pregnancy.

It may be fairly asked, and, no doubt, it will quickly occur to the reader to ask: How do you, then, account for such apparently great disparity in the duration of gestation in cases where only a single act of coitus took place at a known time? From the views. viz., that, first, spermatozoa may reach the uterine cavity within a few hours from coitus held immediately before or after menstruation, and fecundate an ovum which may have descended thereto during the menstrual epoch now imminent or just ended, as would seem to have happened in seven or eight of my cases; and, second, that spermatozoa must ascend nearly or

* Dr. Tyler Smith, *Lancet*, March, 1856, page 333.

even to the ovary, there fecundate an ovum which, in turn, must descend to the uterus: the ascent of the sperm cells and the descent of the impregnated ovum occupying jointly from ten to twenty days or more. These views will, I think, explain the seeming prolongation in the duration of certain cases of pregnancy resulting from a single coitus at a known time. Apparently protracted gestation is certainly more easily and more reasonably explained in this manner than in any other way. In all the cases of supposed protracted gestation, no evidence whatever, beyond a mere assumption, is adduced to prove that conception really took place at the time of the known coitus. Why is it assumed that, in such cases, conception followed immediately on coitus? Might it not so happen that the spermatozoa should travel all the way up to the ovary before meeting an ovum? And might it not also happen that when they did reach the ovary no ovum was matured for perhaps a few days after and was consequently unfit for fecundation? And then the fecundated ovum must come down to the uterine cavity. Take the sixth case of my table: The woman was absent from her husband for fifteen days after the cessation of her last courses. Yet the ovum matured at the last menstrual epoch did not reach the uterine cavity until the 27th of September, the occasion of the fruitful coitus. Were she, however, with her husband at the time of the cessation of her last menses, the duration of pregnancy would be set down at 288 days, instead of 272 days actual time. Besides this case, there is ample evidence to prove that fecundation may not take place for many days after a single known coitus. The whole trouble and error, in my estimation, lies in the absurd assumption, that because a woman conceives after a certain act of intercourse, she does so almost immediately. This is unreasonable.

The duration of pregnancy should be calculated, in my estimation, from the time the impregnated ovum takes up its place in the uterine cavity to the outset of labor. That period I believe to be, as I already said, 275 *days when perfectly normal.* Any case of labor before 275 days I would consider abnormal—premature. And we all know how very easy it it is, sometimes—what seemingly trivial causes will now and then determine the outset

*TABLE OF THE DURATION OF HUMAN UTERO-GESTATION.*

| Case. | Menstruation began. | Menstruation ceased. | Labor began. | Duration of Pregnancy, in days, from end of last menses, or from coitus. | Number and Sex of Child | From beginning of last menses, to Labor, in Lunar months. | From end of last menses, to Labor, in Calendar months. | Remarks. |
|---|---|---|---|---|---|---|---|---|
| 1 | April 3, 1875. | April 6, 1875. | On the evening of Jan. 6, 1876. | 274 | 1, male. | 10 less 2 days. | 9 less 1 day. | Married April 7, 1875, and first coitus took place that night. Believed she conceived from first coitus. |
| 2 | Oct. 7, 1875. | Oct. 10, 1875. | July 13, 1876. | 276 | 1, male. | 10 exactly. | 9 plus 3 days. | |
| 3 | Oct. 17, 1875. | Oct. 20, 1875. | July 23, 1876. | 276 | 3, female. | 10 exactly. | 9 plus 3 days. | |
| 4 | Nov. 19, 1877. | Nov. 22, 1877. | Aug. 25, 1878. | 275 | 1, female. | 10 less 1 day. | 9 plus 3 days | |
| 5 | Feb. 7, 1878. | Feb. 10, 1878. | At 8 o'clock, a. m., Nov. 15, 1878. | 277 | 1, male. | 10 plus 1 day. | 9 plus 4 days. | |
| 6 | Sept. 9, 1878. | Sept. 12, 1878. | June 26, 1879. | 272 | 2, male. | 10 plus 10 days. | 9 plus 14 days. | Was absent from her husband before and at time of menses, and until the night of Sept. 27. |
| 7 | Dec. 12, 1878. | Dec. 15, 1878. | At 1 o'clock a. m., Sept. 14, 1879. | 275 | 3, male. | 10 less 5 days. | 9 less 2 days. | Conception took place just before onset of a partial menstruation. The three days of partial menses are counted. |
| 8 | Dec 31, 1878. | Jan. 3, 1879. | Evening of Oct. 5, 1879. | 275 | 2, male. | 10 plus 2 days. | 9 plus 2 days. | |
| 9 | Jan. 9, 1879. | Jan. 12, 1879. | Evening of Oct. 11, 1879. | 272 | 2, male. | 10 less 5 days. | 9 less 1 day. | |
| 10 | Feb. 5, 1879. | Feb. 8, 1879. | Morning of Nov. 13, 1879. | 277 | 5, female. | 10 plus 1 day. | 9 plus 4 days. | |
| 11 | Feb. 21, 1879. | Feb. 24, 1879. | Morning of Dec. 2, 1879. | 280 | 2, female. | 10 plus 4 days. | 9 plus 8 days. | |
| 12 | Mar. 3, 1879. | Mar. 5, 1879. | Morning of Dec. 6, 1879. | 275 | 3, female. | 10 less 2 days. | 9 plus 1 day. | |
| 13 | April 12, 1879. | April 15, 1879. | Jan. 16, 1880. | 275 | 6, female. | 10 less 1 day. | 9 plus 1 day. | |
| 14 | April 25, 1879. | April 28, 1879. | Feb. 6, 1880. | 280 | 1, female. | 10 plus 7 days. | 9 plus 9 days | Married May 1, 1879, and first coitus took place that night. |

of labor a few days before full term. And any case happening after 276 days, I would consider due to the non-meeting and impregnation of the germ cell by the spermatozoa for some days after coitus.

Any special comments on this table are unnecessary. As a rule the day of labor is not counted in the duration of utero-gestation. But in the few cases where labor set in on the evening of any day, that day is counted in the duration of pregnancy. All of my fourteen cases are intelligent married ladies, in whom the function of menstruation is normal. The outset, duration, and cessation of the catamenia were accurately known in each case. Not one of these fourteen ladies could have the slightest object in deceiving me. The duration of pregnancy was calculated in the presence of each lady, from the data furnished by her for the sole and plain purpose of finding the date of labor. The last day of the menstrual flow, that is, the day it ceased, is not counted in the duration of utero-gestation, because the fruitful coitus did not take place until the night of the day on which the flow ceased. The few cases where menstruation had terminated one or more days before coitus was possible, are noted in the table. These are, it will be seen, cases 1, 6, and 14. Case 7 is positive, from well-known symptoms and sensations, that she conceived from a coitus held just before the outset of the menses, which were not entirely, although very nearly, suppressed. The three days of very slight menstruation are included in the duration of gestation. Her labor began 275 days from the fruitful coitus, and five days before the tenth recurrence of the menstrual epoch. Nine of the fourteen cases show that labor set in, almost to the very day, at a time to correspond with a tenth recurrence of menstruation. But in each of these nine, it is most probable that the coitus which took place the night following the cessation of the menses, was the fruitful one. This accounts for the close coincidence of the two. The remaining five show quite a disparity in time between the two facts; but this difference can be explained easily in cases 6 and 14. Case 14 is a primipara, the child a female; yet, from first coitus to labor is just 280 days. In cases 1, 2, 4, and 5, being primiparæ also, the child, though of different sex, was carried to full term.

SIOUX CITY, Iowa.

